# Lung Ultrasound and Sonographic Subpleural Consolidation in COVID-19 Pneumonia Correlate with Disease Severity

**DOI:** 10.1155/2021/6695033

**Published:** 2021-01-04

**Authors:** Zouheir Ibrahim Bitar, Mohammed Shamsah, OssamaSajeh Maadarani, Omar Mohammed Bamasood, Ali Zouheir Bitar, Huda Alfoudri

**Affiliations:** ^1^Critical Care Unit, Ahmadi Hospital, Kuwait Oil Company, P.O. Box 46468, Fahaheel, Al Ahmadi 64015, Kuwait; ^2^Adan Hospital, Intensive Care Unit, Hadiya, Kuwait; ^3^University of Waterloo, Canada

## Abstract

**Introduction:**

One of the ultrasonic features of COVID-19 pneumonia is the presence of subpleural consolidation (SPC), and the number of SPCs varies among patients with COVID-19 pneumonia.

**Aim:**

To examine the relationship between disease severity and the number of SPCs on admission. *Methodology*. This observational, prospective, single‐center study included patients with suspected COVID-19 infection who had been transferred to the ICU. A specialized intensivist in critical care ultrasound performed lung ultrasound (LUS) and echocardiography within 12 hours of a patient's admission to the ICU. The aeration score was calculated, and the total number of SPCs was quantified in 12 zones of the LUS.

**Results:**

Of 109 patients with suspected COVID-19 pneumonia, 77 (71%) were confirmed. The median patient age was 53 (82–36) years, and 81 of the patients (73.7%) were men. The aeration score and the counts of subpleural consolidation in each zone were significantly higher in patients with COVID-19 pneumonia (*P*=0.018 and *P* < 0.0001, respectively). There was an inverse relationship between PO_2_/FiO_2_, the aeration score, and the number of subpleural consolidations. The higher the number of SPCs, the worse the PO_2_/FiO_2_ will be.

**Conclusions:**

Sonographic SPC counts correlate well with the severity of COVID-19 pneumonia and PO_2_/FiO_2_. The number of SPCs should be considered when using LUS to assess disease severity.

## 1. Introduction

SARS-CoV-2 primarily affects the lower respiratory tract during the early period of infection, causing COVID-19 pneumonia and making imaging diagnosis the early investigation method of choice [1]. Chest X-ray and computed tomography show diffuse bilateral, asymmetric, and patchy lesions located primarily in the periphery of the lung [2].

Normal healthy lungs show pleural sliding and A‐lines (repetitive lines parallel to the pleural line) [3]. The presence of multiple B-lines (more than three lines in one region) indicates interstitial syndrome. B-lines are shining vertical lines perpendicular to the pleural line that extend from it to the edge of the screen, erasing the A-lines [3].

Lung ultrasound (LUS) is an attractive diagnostic test because COVID-19 disease involves the periphery of the lung and because it is a bedside tool with good diagnostic accuracy. LUS has been used for triage in the emergency department (ED) during the pandemic; it informs decisions to allocate patients for proper treatment to avoid unnecessary exposure of other patients and medical staff when patients are allocated to unsupervised areas [4]. LUS is used in COVID-19 pneumonia for disease identification before RT-PCR results, disease severity classification, or treatment allocation [4].

To estimate disease severity by LUS, the total number of SPCs on admission and B-lines, as percentages in each field of the examined chest area, are typically used [5]. To date, the correlation between the number of subpleural consolidations and the severity of COVID-19 pneumonia has not been examined.

## 2. Methods

### 2.1. Study Population

This study was an observational, prospective, single‐center study conducted in the intensive care unit of Adan General Hospital from May 1, 2020, to June 30, 2020. The Ethical Committee of the Ministry of Health in Kuwait approved the study protocol, and informed consent was obtained from every patient or his or her next of kin.

Patients were included if they were aged >18 with suspected COVID-19 infection and had been transferred to the ICU with fever or suspected respiratory infection plus one of the following: respiratory rate >30 breaths/min, severe respiratory distress, or SpO_2_ <93% on room air [6].

Reverse transcription polymerase chain reaction (RT-PCR) was performed on every patient admitted to the ICU. The treating critical care physician entered the clinical data in a separate data collection form at the time of patient enrollment. A specialized intensivist in critical care ultrasound performed the lung ultrasound within 12 hours of the patient's admission to the ICU and saved the data for a second revision by another critical care specialist. The physician conducting the ultrasonography was blinded to the RT-PCR results.

### 2.2. Protocol

We performed lung ultrasonography of every patient admitted to the ICU with suspicion of COVID-19 infection using a 12-zone method [3]. The anatomical landmarks were the parasternal line, anterior and posterior axillary lines, and paravertebral line, demarcating three areas per hemithorax (anterior, lateral, and posterior). The anterior chest wall was defined as extending from the parasternal line to the anterior axillary line. This zone was divided into upper and lower regions at the third intercostal space. The lateral area from the anterior to the posterior axillary line was divided into upper and lower halves. The posterior zone was located between the posterior axillary line and the paravertebral line.

**Figure 1:**
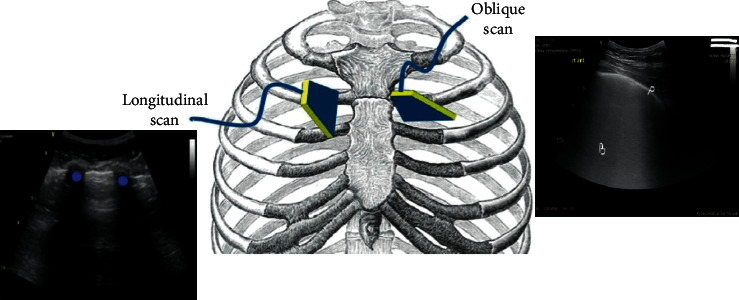
The oblique position of the probe on the intercostal space allows visualization of a larger portion of the pleural line (P) without interruption from the rib shadows (dots) in the longitudinal scan; B, coalesced B-lines.

Ultrasound images were saved to a hard drive and reviewed by a second senior intensivist trained in critical care ultrasound. The interrater agreement was showing high levels of internal consistency (intraclass correlation coefficient (ICC) = 0.95, *P* < 0.001). Ultrasound was performed using a portable ultrasound machine (GE Vivid IQ, Horten, Norway) equipped with a 3.5 MHz broadband curvilinear transducer. A curvilinear transducer was used because it covers more area and allows for visualization and calculation of B-line scores as well as counting of the number of SPCs, although SPCs are more apparent on the linear probe. The probe was placed in an oblique position on the intercostal space, and the pleural line was centered in the middle of the image by adjusting the depth settings. The oblique position of the probe on the intercostal space allowed visualization of a larger portion of the pleural line without interruption from rib shadows (Figure 1).

### 2.3. Measurements

**Figure 2:**
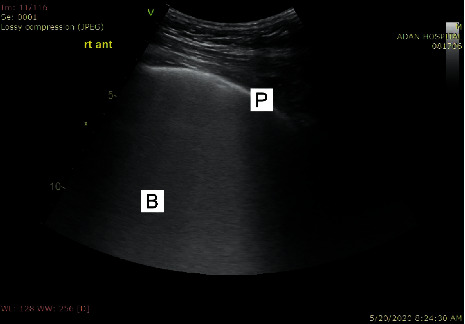
Coalescent B-lines giving the appearance of a shining white lung with irregular pleura. The B-lines maintain their brightness until the end of the screen. P, pleura; B, B-lines.

**Figure 3:**
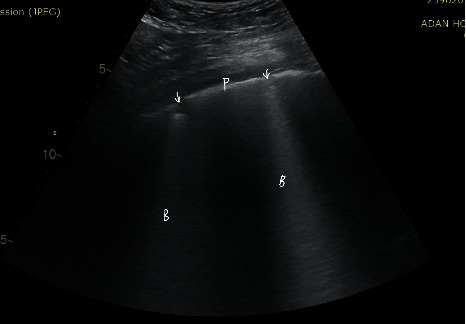
Separated B-lines with irregular pleura. P, pleura; B, B-lines; arrow, subpleural consolidation.

The signs of high probability COVID-19 pneumonia on lung ultrasound evaluation are as follows (Figures 2 and 3):

1. Patchy distribution of multiple coalesced and separated B-lines with the light beam sign, with bilateral and well-demarcated separation from large “spared” areas
2. The pleural is sliding and might appear irregular and fragmented
3. Multiple small subpleural consolidations are limited to the periphery of the lungs

A light beam may be visualized below small peripheral consolidations and zones with irregular pleural lines.

We counted the number of SPCs in each zone and summed the numbers in the 12 chest zones.

For the aeration score, each lung zone was assigned a score to predict overall lung aeration as follows [3]: Score 0: predominant A-lines or <3 separated B-lines; Score 1: at least three B-lines or coalescent B-lines occupying <50% of the screen without an irregular pleural line; Score 1p: at least three B-lines or coalescent B-lines occupying <50% of the screen with an irregular pleural line; Score 2: coalescent B-lines occupying >50% of the screen without an irregular pleural line; Score 2p: coalescent B-lines occupying >50% of the screen with an irregular pleural line; and Score 3: large consolidations (>1 cm).

## 3. Results

Of the 109 patients with suspected COVID-19 pneumonia, 77 (71%) were confirmed. The median patient age was 53 (82–36) years, and 81 of the patients (73.7%) were men. The clinical characteristics of the patients with confirmed COVID-19 pneumonia are shown in Table 1.

**Figure 4:**
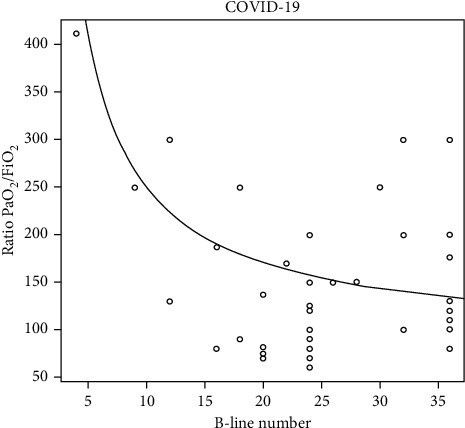
Demonstrates for COVID-19 patients, an inverse relationship between aeration score (B-line number) and the ratio PaO_2_/FiO_2_.

**Figure 5:**
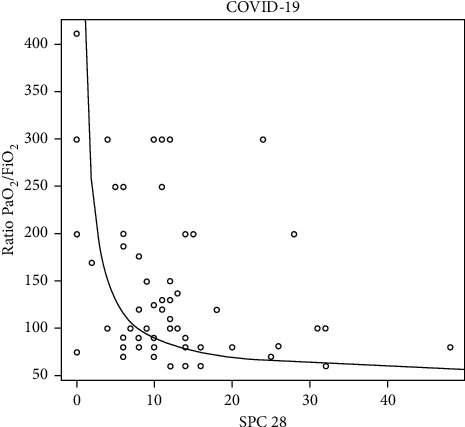
Demonstrates for COVID-19 patients, an inverse relationship between subpleural consolidation (SPC) and the Ratio PaO_2_/FiO_2_.

The aeration score and subpleural consolidations in each zone were significantly higher in COVID-19 pneumonia patients (*P*=0.018 and *P* < 0.0001, respectively). There was an inverse correlation between PO_2_/FiO_2_, the aeration score, and the subpleural consolidation number. The higher the number of SPCs, the lower the ratio will be (Figures 4 and 5).

LUS revealed signs of a high probability of COVID-19 pneumonia in 75 patients (97.4%) (sensitivity 96.9%; CI 85%–99.5%). Two patients presented with unilateral lobar pneumonia without other ultrasonic signs of COVID-19 pneumonia but had positive RT-PCR results. Among the patients without COVID-19 pneumonia with negative RT-PCR results, 32 (90%) were LUS-negative for COVID-19 pneumonia (specificity 91.7%; 95% CI 58.72%–99.77%).

## 4. Discussion

Ultrasonic imaging of COVID-19 pneumonia shares common features with other viral pneumonias, but the presence of morphologically distinct features of subpleural consolidation may represent a major feature of lung involvement, drawing into question the true nature of the consolidative lesions. These distinct features reveal that COVID-19 comprises an immune response induced by the infection that is different from other viral pneumonias [7].

Small studies have attempted to explain the nature of subpleural consolidation. Soldati et al. studied 3 COVID-19 patients with pneumonia using contrast-enhanced ultrasound. The net findings were suggestive of consolidations with perfusion defects, which could be caused by ischemic or necrotic changes that differ from other causes in consolidations, such as inflammatory or atelectasis events [8]. Zotzmann et al. selected 3 out of 10 SARS-CoV-2-positive patients with a medium to high risk of pulmonary embolism on mechanical ventilation [9]. The patients had elevated D-dimer levels and mild to moderate pulmonary hypertension on prophylactic heparin and were assessed for the presence of multiple subpleural consolidations. The 3 cases presented with segmental pulmonary embolism, as proven by chest contrasted computed tomography [9]. Another case series, using contrast-enhanced ultrasound imaging to study SPCs, demonstrated perfusion defect areas demarcated with respect to the perfused lung parenchyma. The consolidations with perfusion defects observed in those patients could be supported by ischemic and necrotic changes in addition to inflammatory or atelectasis events. The same patients exhibited revascularization of these defects upon recovery [10]. These findings may have importance in understanding the pathogenesis of the consolidations in COVID-19 and, consequently, in the medical management of patients with COVID-19 pneumonia.

Counting B-lines is the primary factor in determining the severity of disease by LUS, with the aeration score being the most commonly used metric [11]. Aeration is classified in each lung zone from a score of 0 (predominant A-lines) to a score of 3 (large consolidations (at least >1 cm)) [11]. In contrast, the exact number of B-lines is difficult to determine, as it depends on the transducer used and the interpretation of the rater [12]. Technical factors, frequency, image quality, transducer width, and therefore, the area of lung tissue visualized can impact the B-lines interpretation. Correct interpretation of B-lines influences both the prognosis and clinical diagnosis.

As consolidation in COVID-19 pneumonia exhibits differential pathology and distribution, we opted to count the SPCs in each lung zone as an indication of severity. The relationship between PaO_2_/FiO_2_ and the number of SPCs indicates that our findings are important. Knowing the number of subpleural consolidations could help determine the severity and prognosis of the disease rather than depending only on B-line counting and/or the presence of large consolidations that are rarely found in COVID-19 pneumonia.

This study has certain limitations, including the sample size. This study included two experienced ultrasonographers; however, whether the results would differ based on experience/operator is unclear. In addition, whether the number of SPCs and the course affect prognosis requires future studies with larger sample sizes.

## 5. Strengths and Limitations

1. LUS can be used in COVID-19 pneumonia for disease identification and disease severity classification.
2. Disease severity classification by LUS depends mainly on the percentage of B-lines in each chest zone. SPCs are ultrasonic features of COVID-19 pneumonia and vary in number among patients.
3. Sonographic SPC numbers correlate well with the severity of COVID-19 pneumonia and PO_2_/FiO_2_.
4. Study limitations include being a single-center study.

## 6. Conclusion

In conclusion, the nature and site of SPCs are unique in COVID-19 pneumonia. The number of SPCs correlates with disease severity and should be considered when scoring disease severity rather than depending only on B-line percentages.

## Figures and Tables

**Table 1:** Clinical characteristics of the patients.

	Confirmed COVID-19, 77 cases (71%)	Non-COVID-19, 32 cases (29%)	Total 109 cases (%)	*P* value
Median age (IQR) (years)	53 (82–36)	68 (25–80)	—	—

Male sex no. (%)	64 (83)	16 (50)	80 (73)	0.005

Medical history no. (%)				
IHD	9 (11)	20 (66)	19 (20)	<0.0001
CABC	2 (2.6)	25 (53)	10 (10)	0.001
Hypertension	20 (25)	12 (80)	32 (34)	0.002
Diabetes mellitus	25 (32)	30 (100)	39 (42)	<0.0001
CVA	8 (1)	2	—	0.3
COPD	3 (3)	3 (20)	2 (2)	0.02
Chronic renal impairment	25 (32)	22 (66)	10 (66)	0.001
Hemodialysis	6 (0.7)	9 (25)	—	0.01
Cancer	0	1 (6)	1 (1)	0.091

Status on admission to ICU no. (%)				
Acute MI	2 (2.6)	20 (10)	12 (13)	0.001
Acute PE	9 (11.6)	0	—	0.038
Acute CVA	6	0	—	0.117
Symptom duration (median, days)	5 (2–10)	2 (3–4)	—	—
Hypoxemia	All	All	109 (100%)	0.02
PO_2_/FiO_2_ (mean)	145	226	—	0.026
HFNC	35 (71)	2 (33%)	50 (64%)	0.002
IV	21 (27)	7 (26%)	25 (27%)	—
Facemask	17 (22)	2 (13%)	19 (20%)	—
SOFA score (mean)	7.7	6	—	0.39
RT-PCR positive (%)	75 (97.4)	0	—	—
RT-PCR negative (%)	2 (2)	32 (100)	—	—

US chest finding				
Aeration score (mean)	27 ± 9	21 ± 6.5	0.018	—
SPC score (mean)	13 ± 10.7	1 ± 0.258	<0.0001	—

Plus-minus values are means ± SD; IHD, ischemic heart disease; HFNC, high-flow nasal cannula; IV, invasive ventilation; COPD, chronic obstructive pulmonary disease; SPC, subpleural consolidation.

## Data Availability

The data supporting the findings of this study are available from the corresponding author upon request.

## Additional Points

Patients and/or the public were not involved in the design, conduct, reporting, or dissemination plans of this research. Patient consent for publication was not required.
